# Children’s Environmental Health in Thailand: Past, Present, and Future

**DOI:** 10.29024/aogh.2301

**Published:** 2018-08-31

**Authors:** Ratchaneewan Sinitkul, Chathaya Wongrathanandha, Somkiat Siriruttanapruk, Adisak Plitponkarnpim, Richard J. Maude, Emma L. Marczylo

**Affiliations:** 1 Faculty of Medicine Ramathibodi Hospital, Mahidol University, Bangkok, TH; 2 Bureau of Occupational and Environmental Disease, Ministry of Public Health, Bangkok, TH; 3 Toxicology Department, Centre for Radiation, Chemical and Environmental Hazards, Public Health England, Chilton, UK; 4 Mahidol-Oxford Tropical Medicine Research Unit, Faculty of Tropical Medicine, Mahidol University, Bangkok, TH; 5 Centre for Tropical Medicine and Global Health, Nuffield Department of Medicine, University of Oxford, Oxford, UK; 6 Harvard TH Chan School of Public Health, Harvard University, Boston, US

## Abstract

**Background:**

There is increasing evidence of a link between environmental pollution and preventable diseases in developing countries, including Thailand. Economic development has generated several types of pollution that can affect population health. While these environmental health effects can be observed throughout life, pregnant women and children represent particularly vulnerable and sensitive groups.

**Methods:**

The published epidemiological literature investigating environmental chemical exposure in Thai children was reviewed, highlighting those that investigated associations between exposure and subsequent health outcomes.

**Results:**

The majority of the Thai epidemiological studies on environmental health in children were cross-sectional in design, with some demonstrating associations between exposure and outcome. The three main types of chemical exposure in Thai children were pesticides, heavy metals, and air pollution, which resulted from agricultural activities in countryside areas, industrial zones (both registered and unregistered establishments), mining, and traffic in inner cities. Major health outcomes included detrimental effects on cognitive function and cancer risk. Pesticide exposure was focused on, but not limited to, agricultural areas. The success of the Thai environmental policy to introduce lead–free petrol can be demonstrated by the decline of mean blood lead levels in children, particularly in urban areas. However, unregistered lead-related factories and smelters act as hidden sources. In addition, there is increasing concern, but little acknowledgement, about the effects of chronic arsenic exposure related to mining. Lastly, air pollution remains a problem in both dense city populations due to traffic and in rural areas due to contamination of indoor air and house dust with heavy metals, endotoxins and other allergens.

**Conclusions:**

The increasing number of published articles demonstrates an improved awareness of children’s environmental health in Thailand. Chemical hazards, including the improper use of pesticides, environmental contamination with heavy metals (lead and arsenic), and air pollution in inner cities and indoor air, continue to be growing issues.

## Introduction

According to the epidemiological literature, there is increasing evidence of an association between environmental pollution and chronic non-communicable diseases (NCDs) in developing countries, including Thailand. The World Health Organization (WHO) estimated in 2012, the global prevalence of environmental health diseases at approximately 26% of childhood death, mainly affecting developing countries such as Thailand (19% prevalence in the Thai population) [1]. Urbanization, globalization, and industrialization appear to be the main contributors to the transition from infectious to chronic non-communicable diseases in developing countries [2].

### Thailand at a Glance

Thailand consists of four regions: Northern Thailand, Northeastern Thailand, Central Thailand, and Southern Thailand. Each region has its own unique historical background, geology, and culture that affect the different types of pollution and patterns of disease. However, over the past 20 years, environmental hazards across the country have been changing from traditional threats, such as bacterial contamination of drinking water and wood smoke in poorly ventilated residences, to new environmental threats, such as traffic-related emissions; asbestos construction materials; untreated manufacturing waste; electronic appliance waste; heavy metal contaminants such as lead, arsenic, cadmium from anthropogenic activities such as mining; pesticides; and persistent organic pollutants (POPs). Since 2011, The World Bank has categorized Thailand as an upper-middle income economy based on gross national income (GNI) per capita, with the country’s major income source changing from agriculture to the industrial sector [3]. However, economic development via industrialization and globalization is increasing at a greater rate than environmental preservation. This has generated several types of pollution that can affect population health. Health effects can occur throughout life; however, pregnant women and children represent particularly vulnerable and sensitive groups [2].

The main ministry for pollution control is the Ministry of Natural Resources and Environment, especially the Department of Pollution Control. From a public and environmental health policy perspective, the challenges to children’s environmental health in Thailand include the lack of efficiently regulated controls and the reluctance to recognize and/or use the precautionary principle. The environmental legislation in Thailand is based on proof of evidence. In addition, awareness of environmental health problems is relatively limited among health care providers as well as the general population. Compared to 20 years ago, however, the approach of the health care system towards environmental problems has improved. The positive direction of public policy and research in children’s environmental health in Thailand includes several international collaborations that reflect increasing awareness and the opportunity to exchange knowledge on environmental health. Both government agencies and non-governmental organizations such as Ecological Alert and Recovery–Thailand (EARTH) are involved in coordinating such initiatives. As a result, research is increasing, particularly as advances in technologies for the detection, monitoring, and intervention of exposures are made. Furthermore, collaboration could augment harmonization of methods and results that are very useful to evaluate uncommon outcomes such as cancer. Multidisciplinary government inter-departmental and agency collaboration is essential to help protect Thai children from unhealthy environments.

### Hazard Identification: Physical, Biological, and Chemical

The catastrophic tsunami in 2004, which killed 230,000–280,000 people in 14 countries, including Thailand, was one of the most tragic environmental emergency disasters. Flooding and drought are also increasing, with possible links to climate change and global warming. Radiation exposures (environmental and medical) receive less attention in the literature from Thailand. However, there is concern over radon exposure in specific geographical locations [4,5]. and also about radiation used for medical diagnostics and interventions for some pediatric patients [6,7]. Thus, in combination with the multiple pollutants already mentioned as a result of urbanization, globalization and industrialization, there is a wide variety of environmental exposures relevant to children’s environmental health in Thailand. For the purposes of this review, we limited exposure to environmental chemicals and air pollution, exposures most relevant to children in Thailand.

## Objectives

This review aims to 1) conceptualise children’s environmental health in Thailand through a comprehensive review of the available epidemiological literature investigating environmental chemical exposure in Thai children, and 2) propose the future direction of research, policy, and practice to improve the health of children in Thailand and similar upper-middle income countries.

## Methods

We searched the health research databases on children’s environmental health in Thailand in the Medline/PubMed, Web of Science, EMBASE, and Scopus databases using the PICO (Problem/Patient/Population, Intervention/Indicator, Comparison, Outcome, and (optional) Time element or Type of Study) search strategy shown in Table 1. Randomized controlled, retrospective, prospective, case-controlled, and cross-sectional studies, and case reports on human cohorts in Thailand from 1980–2017 were included. Literature published in Thai and/or English was included to minimize selection bias from language barriers. Articles were excluded if they: 1) did not meet the inclusion criteria, 2) focused on physical hazards including radiation, electromagnetic field, noise, heat, cold, or injury 3) focused on psycho-social hazards including stress, poverty, or inequity, and 4) focused on biological hazards (except aeroallergens; mold, fungi, and cockroaches) such as bacteria, viruses, insects, plants, birds, or animals. Aeroallergens were included due to the link with chronic respiratory symptoms.

**Table 1:** Main concept and alternative terms of PICO search strategy.

	Main concept	Alternative terms

**Population**	**Thai**	Thailand
	**Child??**	Kid?, Pregnan*, “in utero”, newborn, neonat*, infan*, toddler, preschool, young, teenage*, pupils, student?
**Exposure**	**Environment**	Chemical, Pollution?, “Air quality”, PM, “particulate matter”, particle, VOCs, “volatile organic compounds”, benzene, “polyaromatic hydrocarbon”, PAH, allergen, mold, mould, fungi, cockroach, smok???, metal?, arsenic, lead, cadmium, mercury, plasticizer, POPs, “persistent organic pollutants”, “Polychlorinated biphenyl”, Dioxins, Furans, “Polybrominated compounds”, “Polyfluorinated compounds”, “Halogenated hydrocarbons”, pesticide?, herbicide?, fungicide?, organophosphate, organochlorine, carbamate, glyphosate, chlorpyrifos, Dichlorodiphenyltrichloroethane, phthalate, BPA, “bisphenol A”, Industr*, agricultur*, waste, Hazard???, Toxic
**Health Outcomes**	**Perinatal**	“Birth weight”, prematurity, “birth defects”
	**Growth**	Weight, height, BMI, “body mass index”, obes*
	**“Neurodevelopmental disorders”**	Development*, autistic?, autism, attention, hyperactive, ADHD, learning, cognitive, intelligen*, IQ, aggressive, pervasi*, Behavior*, Behaviour*
	**Allergy**	atop??, inflammation, sensitization, IgE, hypersensitivity, “food allergy”, “atopic dermatitis”, “allergic dermatitis”, wheezing bronchitis, bronchiolitis, hyperresponsive????, hyper-responsive????, asthma, “allergic rhinitis”, “perennial rhinitis”, hayfever, “hay fever”
	**Respiratory**	“pulmonary function”, “Lung function”, “respiratory symptom?”, “respiratory infection”
	**Endocrinology**	Thyroid, diabetes, DM, metabolic?
	**“Cardiovascular diseases”**	Coronary, hypertension, “blood pressure”, Atheroscler*
	**“Kidney diseases”**	Renal, “kidney function”, “renal function”, creatinine
	**“Liver diseases”**	“Liver diseases”, cirrhosis, jaundice
	**Poisoning**	“acute poisoning”, “chronic poisoning”
	**Cancer**	Malignancy, Leukaemia, Leukemia, Lymphoma, Carcinoma
	**Subclinical**	“DNA damage”, epigenetic, miRNA, Histone, Methylation, Adduct

Population (P) = Thai children, Indicator (I) = Exposure (environmental chemical, metal, allergen, or air pollution), Comparison (C) = no comparison, Outcome (O) = Health outcomes. Both * and/or ? were applied to widen the search result by including various word endings and spellings (* = unlimited character number, ? = limited character number (to number of ?s)).

All article titles and abstracts (where appropriate) were screened by two independent reviewers (RS and CW). A third independent reviewer (EM) provided a final decision on the inclusion/exclusion of articles where the first two reviewers had disagreed. Thus, the final list of studies for review was selected in agreement by at least 2 out of 3 independent reviewers (RS, CW, EM). The full text of Thai articles was retrieved from *Journal of Medical Association of Thailand* and *Thai Pediatric Journal*. All available full-text articles were critically reviewed to identify the major environmental chemical and air pollution threats to children living in Thailand, highlighting those that investigated associations between exposure and subsequent health outcomes. Relevant studies cited in the selected studies were also included as appropriate.

## Results

**Figure 1:**
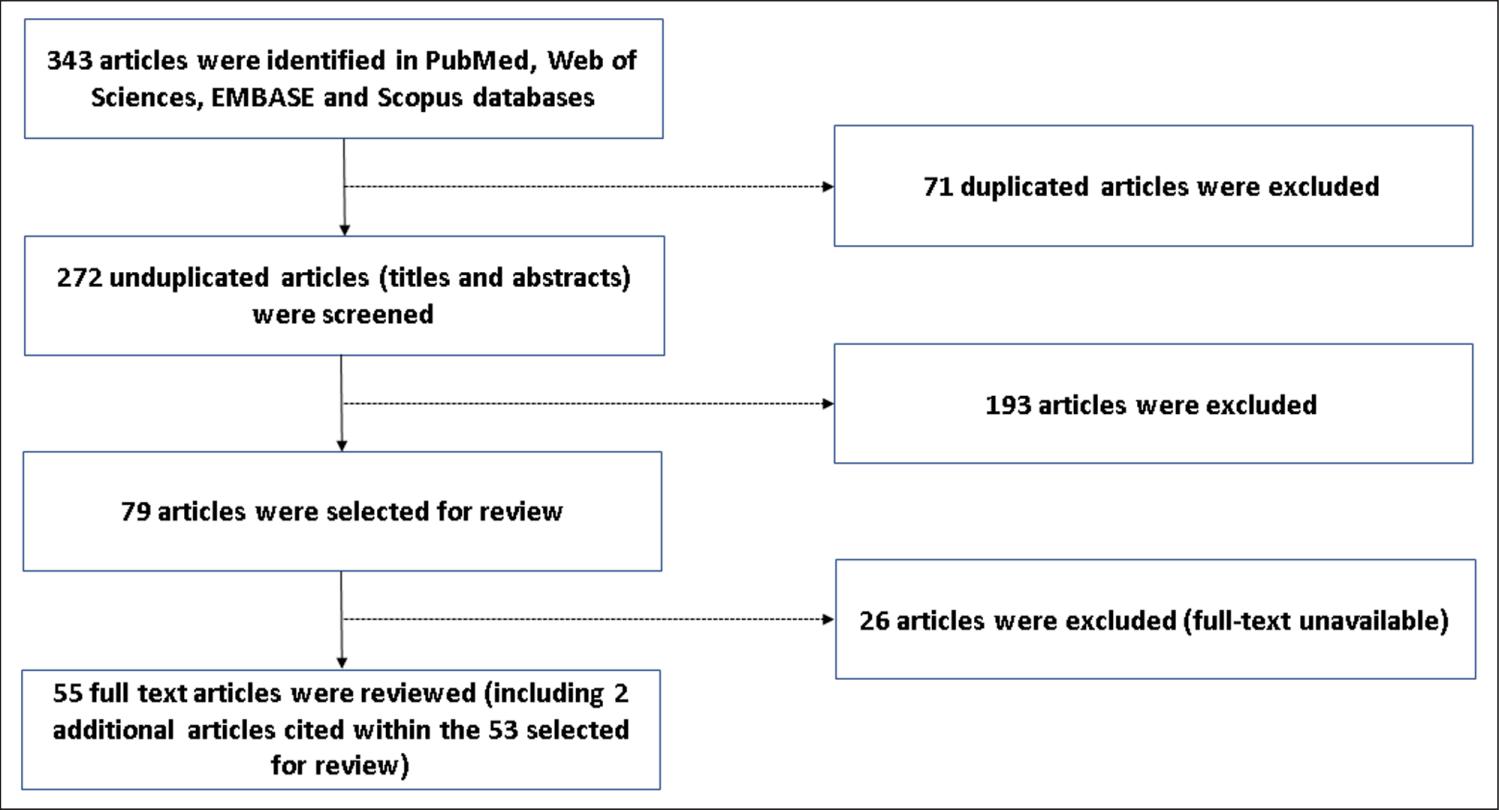
The article selection process.

As shown in Figure 1, 343 articles were identified from four databases, resulting in 273 unduplicated articles. Following screening of titles and abstracts, 79 articles were selected for critical review. Of these 79 articles, 26 were further excluded due to restricted access to the full paper. An additional two articles, which were cited in the key article of arsenic exposure, were included later. Therefore, a total of 55 full-text articles were critically reviewed and summarized in Tables 2, 3, and 4. The majority of exposures were pesticides (organochlorine, organophosphate, paraquat, glyphosate, or paraquat), heavy metals (lead, arsenic, cadmium, or mercury), or air pollution (ambient air pollution or environmental tobacco smoke). The sources of these exposures varied by geographical region.

**Table 2:** Epidemiological studies investigating pesticide exposure in Thai children.

Pesticides	Authors (published year)	Type of epidemiological study	Population (age)	Sample size	Exposure assessment	Level measured	Comments

**Exposure studies**							

**Chlorpyrifos**	Panuwat et al. (2009) [15]	Cross-sectional	Student (12–13 years)	207	Urinary 18 specific pesticide metabolites	14 metabolites of chlorpyrifos, permethrin, pyrethroids were found	4 groups of parental occupations (farmers, merchants and trader, government and company employees, and laborers) Farmers group: significantly higher urinary pyrethroid metabolites levels
**Glyphosate**	Kongtip et al. (2017) [18]	Birth cohort	*in utero* 19–35 years of 28 weeks pregnancy and neonate	82 pairs	Maternal and cord blood serum glyphosate levels Interviews (questionnaire)	Median glyphosate levels in the pregnant women’s serum were 17.5 ng/mL (0.2–189.1 ng/mL), significantly higher than those in umbilical cord serum (median 0.2 (0.2–94.9 ng/mL)	Factors for glyphosate exposure were working in agriculture or living in families that work in agriculture.
**Organophosphate**	Hanchenlaksh et al. (2011) [20]	Cross-sectional	Preschool and school child (2–12 years)	16 Part of larger study that included adults	Urinary DAP metabolites (DEP, DETP, DEDTP, DMP and DMTP)	Metabolites Geometric mean of DAP levels was 7.6 mg/g.	Exposure for farmers’ families seems to be through transfer from the farmer to family members or contamination of the home environment
	Liu et al. (2015) [14]	Cross-sectional	Preschool and school child (2–12 years)	72 Part of larger study that included adults	Urinary DAP metabolites^%^ Self-administered questionnaires	Metabolites Geometric mean of DAPs levels was 3.12 μg of creatinine	Children in Thai farming families can be exposed to pesticides indirectly through transfer of pesticide residues from the farmer to the spouse and subsequently to the child, or through contamination of the home environment.
	Panuwat et al. (2009) [16]	Cross-sectional	School child (10–15 years)	306 Part of larger study that included adults	Urinary parathion metabolites (PNP, DMP, DMAP, DMTP)	Metabolites Geometric mean of PNP, DMP, DMTP, and DMAP were 3.5 ng/mL, 1.9 ng/mL, 1.6 ng/mL and 29.9 nmol/mL, respectively	25–60% of the PNP (metabolites of **^#^parathion**) detected among farmers and children
	Petchuay et al. (2006) [13]	Case-controlled	Preschool (2–5 years)	54 (farm: 37, rubber plantation: 17)	Urinary DAP metabolites (DMP, DEP, DMTP, DETP, and DEDTP)	Metabolites	Farm children tended to have significantly higher DAP concentrations compared with children living outside the farming area, particularly during the dry vegetable-planting season.
	Rohitrattana et al. (2014) [19]	Cohort	*in utero* (20–35 years of 28 weeks pregnancy until 2 months post-partum)	86, 87, 51 (28 weeks pregnancy, delivery, 2 months post- partum)	Urinary DAP metabolites (DMP, DEP, DETP, DEDTP, total DEP, and DAPs) Interviewed questionnaires (agricultural activities)	Metabolites: 50th percentile of DMP, DEP, DETP, DEDTP, total DEP, DAPs (nmol/L: *nmole/g creatinine*) 28 weeks pregnancy 37.7:*48.9*, 14.6:*20.8*, ND:*ND*, ND:*ND*, 45.2:*84.1*, 90.1:*160.9* Delivery 36.2:*50.5*, 14.4:*18.8*, 2.4:*6.5*, 2.3:*4.5*, 64.9:*87.4*, 108.9:*190.8* 2 months postpartum 38.0:*25.6*, 17.3:*14.8*, ND:*ND*, ND:*ND*, 46.7:*56.1*, 92.1:*101.8*	The levels of urinary OP metabolites at 28 weeks, delivery, and 2 months postpartum fluctuated depending on their pesticide exposures both at home and agricultural activities.
**Paraquat**	Kongtip et al. (2017) [18]	Birth cohort	*in utero* 19–35 years of 28 weeks pregnancy and neonate	82 pairs	Maternal and cord blood serum paraquat levels Interviews (questionnaire)	Median paraquat levels in pregnant women’s serum were <0.4 ng/mL (0.2–58.3 ng/mL) and similar to those in umbilical cord serum (median 0.2 (0.2–47.6 ng/mL)	Factors for paraquat exposure were work in agriculture or live in families that work in agriculture.
**Permethrin Pyrethroids**	Panuwat et al. (2009) [15]	Cross-sectional	Student (12–13 years)	207	Urinary 18 specific pesticide metabolites	14 metabolites of chlorpyrifos, permethrin, pyrethroids were found.	4 groups of parental occupations (farmers, merchants and trader, government and company employees, and laborers) Farmers group: significantly higher urinary pyrethroid metabolites levels
**Pyrethroid**	Rohitrattana et al. (2014) [17]	Case-controlled	School child (6–8 years)	53 (Case 24, controlled 29)	Urinary PYR metabolites (ie, 3-PBA DCCA) Hand wipe samples Structured-interviews	Median urinary PYR metabolites were not significantly between both group, neither wet nor dry seasons.	Positive correlation (r = 0.40–0.46) between PYR residues collected from the hands and urinary PYR metabolites PYR use in rice farms and households may be significant sources of PYR exposure among children living.

**Exposure and health outcome assessment studies**							

**^$^Organochlorine**	Asawasinsopon et al. (2006) [8]	Cross-sectional	*in utero*	39 pairs	Maternal and cord blood serum DDT metabolites	p,p’-DDE) was the highest level in both maternal and cord serum (geometric mean of 1,191 ng/g lipids in maternal serum and 742 ng/g lipids in cord serum)	Negative association between cord serum total T4 levels and DDT metabolites
**Organophosphate**	Fielder et al. (2015) [11]	Case-controlled	School child (6–8 years)	53 (case 24, controlled 29)	Urinary metabolites of OPs (6 DAP^%^, TCPy)	Rice farm had significantly higher total DAP and TCPy	No significant adverse neurobehavioral effects were observed between participant groups during either the high or low pesticide use season
	Kongtip et al. (2017) [12]	Birth cohort	*in utero*	50 pairs (20–35 years of pregnant women and 5 months old of their child)	Maternal urinary DAP metabolites: DMP, DEP, DETP, DEDTP	Median adjusted urinary DAP levels of 28 weeks gestational age of pregnant women were 36.83, 15.15, 0.07, and 0.15 nmol/L for DMP, DEP, DETP, and DEDTP, respectively.	Higher total DEP and total DAP metabolite level from the 28 weeks GA of pregnant women were significantly associated with reduced cognitive and motor composite scores on the Bayley-III at five months old of their child.
**Pyrethroid**	Fielder et al. (2015) [11]	Case-controlled	School child (6–8 years)	53 (case 24, controlled 29)	Urinary metabolites of PYR (DCCA)	Rice farm had significantly higher DCCA	No significant adverse neurobehavioral effects were observed between participant groups during either the high or low pesticide use season

**^#^Parathion** was banned in 2007; data was collected in 2006. **^$^Organochlorine** was banned in early 1983; data was collected in 2003–2004. **^%^Common 6 DAP metabolites**: DMP, DEP, DMT, DMDTP, DETP, and DEDTP. **Abbreviations**: DAP: dialkyl phosphate, DCCA: cis/trans-2,2-(dichloro)-2-dimethylvinylcyclopropane carboxylic acid, DDT: dichlrodiphenyltrichloroethane, DEDTP: diethyl dithiophosphate, DEP: diethyl phosphate, DETP: diethyl thiophosphate, DMAP: dimethylalkylphosphate, DMDTP: dimethyldithiophosphate, DMP: dimethylphosphate, DMTP: dimethylthiophosphate, OPs: organophosphates, PNP: paranitrophenol, p,p’, DDE: 1,1-dichloro-2,2-di(4-chlorophenyl)ethylene, PYR: pyrethroid, TCPy: 3,5,6-trichloropyridinol, 3-PBA: 3-phenoxybenzoic acid.

**Table 3:** Epidemiological studies investigating heavy metal exposure in Thai children.

Heavy metals	Authors (published year)	Type of epidemiological study	Population (age)*	Sample size	Exposure assessment	Level measured	Comments

**Exposure studies**							

**Cadmium**	Chaiwonga et al. (2013) [39]	Cross-sectional	School child (9–12 years and 13–15 years)	748	Urinary cadmium Questionnaires	All urine samples had cadmium of more than 1 μg/g creatinine (Thai general population is 0.5 μg/c Cr) with 2% had higher than 5 μg/g Cr.	The likely exposure sources was dietary (approximately 20–50% of them consume home-grown rice)
**Lead**	Chomchai et al. (2005) [30]	Case**-**controlled	Infant and preschool child (6 months–4 years)	296 (exposure = 114, controlled 149)	Blood lead Questionnaires	Average BLL was 5.65 ± 3.05 μg**/**dL. The overall prevalence of children with EBLL > 10 μg**/**dL was 8.1%, while that of the Klong Toey community was 12.5%.	Predictors factors of EBLL: peeling paint, eating paint chips, and geographical location Saliva was not suitable for the biomarker of lead exposure
	Maharachpong et al. (2006) [31]	Cross**-**sectional	Preschool child to adolescent (4–14 years)	319 (from 242 households)	House dust and soil lead level spatial distribution)	Mean house dust lead was 210 mg/kg (<ATSDR concerning and a dust-lead standard for London (500 mg/kg)).	The lead content level in soil was exponentialy declined with distance from boat-repair yards (point source contamination)
	Mitchell et al. (2012) [43]	Cross-sectional	Child (6 months–14 years)	642	Capillary blood lead Questionnaires	5.1% had EBLL (cBLL ≥ 10 μg/dL) with highest prevalence in children younger than 2 years.	The risk factors of EBLL were anemia (Hb < 10 g/dL), exposure to car batteries, and taking traditional medicines
	Neesanan et al. (2011) [21]	Retrospective study	Preschool child (3–7 years)	213	Blood lead Interviewed the primary care providers (identify risk factors of EBLL)	Mean BLL was 7.71 ± 4.62 μg/dL (3–25 μg/dL) 26% had BLL ≥ 10 μg/dL.	Risk factors of EBLL were male gender and and source of drinking water from either tap or canal
	Ruangkanchanasetr et al. (1999) [32]	Cross-sectional	Infant to school child	511 (infant 84, preschool child 60, school child 377)	Blood lead Questionnaires	Mean BLL 5.57 ± 2.31 μg**/**dL, 4.75 ± 3.25 μg**/**dL, 6.72 ± 2.02 μg**/**dL, and 9.03 ± 3.65 μg**/**dL at birth, 2 years, kindergartens, and secondary school respectively. EBLL were 1–35%.	Mean BLL and prevalence of EBLL (>10 μg/dL) increased with age Risk factors of EBLL: older age group, larger family size, and male gender
	Swaddiwudhipong et al. (2013) [22]	Cross-sectional	Child (1–14 years)	254	Blood lead Questionnaires Lead environmental survey: water, vegetables grown in the area	Mean BLL was 9.8 ± 5.1 μg/dL. The overall prevalence of EBLL (≥10 μg/dL) was 43.3%	Lead contamination was found in house floor dust, drinking water kept in household containers. 50.8% of EBLL children lived in vented lead-acid batteries, while 23.3% lived in the house without vented lead-acid batteries.
	Swaddiwudhipong et al. (2014) [44]	Cross-sectional	Child (1–14 years)	695	Blood lead Questionnaires Lead environmental survey: floor dust, drinking water, metal pot	Geometric mean BLL was 9.16 μg/dL. Overall prevalence of EBLL was 47.1%	The metal pots were safe for cooking rice but might be unsafe for acidic food preparation.
	Thaweboon et al. (2005) [24]	Cross**-**sectional	Preschool child (3–6 years)	8 Part of larger study that included adult	Salivary and blood lead	All children subjects had BLL > 10 μg/dL The geometric mean for BLL was found to be 24.03 µg/dl (11.80–46.60 µg/dl), lead in saliva was 5.69 µg/dl (1.82–25.28 µg/dl).	Saliva was not correlated with BLL (γ =–0.025).
	Untimanon et al. (2011) [42]	Case-controlled	Child (<15 years)	24 (case 22, controlled 2) Part of larger study that included adult	Lead at on skin(wipe) Household floor and dust lead content Questionnaire-based interview	Mean lead contamination in case vs. controlled Household floor loading: 109.9 vs. 40.1 μg/m^2^ Household dust content: 434.8 vs. 80.8 μg/g Hand loading: 64.4 vs. 36.2 μg/m^2^ Foot loading: 77.8 vs. 43.8 μg/m^2^	Hand lead loading in children was higher than adults Skin lead levels were elevated in family members living in a lead-exposed worker’s house and were related to the levels of household lead contamination.

**Exposure and health outcome assessment studies**							

**Arsenic**	Hinhumpatc et al. (2013) [35]	Case-controlled (follow-up study)	School child (5–8 years)	60 (case 40, controlled 20)	Arsenic in toenails, fingernails, saliva, and urine Salivary and urinary 8-OHdG (DNA damage) and *hOGG1* (DNA repair capacity)	The DNA damage (salivary and urinary 8-OHdG) was increase but DNA repair capacity (hOGG1) was decreased in exposed group,	A follow-up study of prenatally arsenic exposure children and continuing exposure until the age of 5–7 years with matched case controls Potential risk for mutation and cancer from biomarkers.
	Intarasunanont et al. (2012) [45]	Case-controlled	*In utero* and *in vitro* (newborn)	71 (case 55, controlled 16)	Arsenic in drinking water Arsenic in cord blood, nails, and hair *In vitro*: human lymphocytes treated with arsenic DNA methylation in cord blood lymphocyte and *in vitro* lymphocyte treated with arsenic Arsenic in drinking and non-drinking water	Arsenic levels in case vs. controlled were – Cord blood: 5.79 ± 0.5 vs. 1.97 ± 0.64 μg/g – Toenails: 1.52 ± 0.38 vs. 0.12 ± 0.04 μg/g – Fingernails: 1.91 ± 0.38 vs. 0.08 ± 0.05 μg/g – Hair: 0.05 ± 0.01 vs. 0.01 ± 0.003 μg/g.	*In utero* arsenic exposure affects DNA methylation, particularly at the p53 promoter region, which may be linked to the mechanism of arsenic carcinogenesis.
	Phookphan et al. (2017) [36]	Case-controlled (follow-up study)	School child (6–9 years)	81 (case 40, controlled 41)	Arsenic in toenails	Arsenic in toenails in exposed and unexposed children was 8.08 ± 1.47 μg/g and 0.76 ± 0.14 μg/g, respectively.	A follow-up study from arsenic exposure *in utero* with continued exposure throughout early life [45]
	Vitayavirasak et al. (2005) [34]	Case-controlled	School child (10 years)	130 (high exposure area 50, low exposure area 50, controlled 30)	Urinary iAs and its metabolites (MMA, DMA) Questionnaire-based interview Arsenic environmental monitoring: surface soil, ambient air, drinking water, fruit, vegetables and meat	Geometric mean of iAs + metabolites were 54.21, 36.61, and 17.50 μg/g creatinine in high exposure, low exposure and control group, respectively. Average of arsenic in agricultural product was within standard acceptance of US FDA at 2 mg/kg. except the freshwater snail (*Sinotaia ingallsiana*)	Source of exposure was one of food chain (fresh water snail), water (surface and ground) and soil Individual risk behaviors of exposure were soil playing and without hand washing before eating. Probable risk of developing cancer (between 10^–5^ –10^–6^)
**Cadmium**	Swaddiwudhipong et al. (2015) [37]	Case-controlled	School child (7–12 years)	594 (case 301, controlled 293)	Urinary cadmium Questionnaire Urinary β2-MG and calcium	19% of children in this study had urinary cadmium ≥ 1 μg/g creatinine, which was higher in girls and in those consuming rice grown in cadmium-contaminated areas.	Significantly higher geometric mean levels of urinary excretion of β2-MG and calcium (early renal effects) were found among children in contaminated areas compared to those in comparison (non-contaminated) areas
**Lead**	Pusapukdepob et al. (2007) [29]	Cross-sectional	Child (0–15 years)	126 (case 89, controlled 37) Part of larger study that included adult	Lead in ‘soil vegetables’ and meat Lead in blood and teeth	Mean BLL in children of case and controlled groups were 25.75 ± 13.89 μg/dL and 7.68 ± 2 μg/dL, respectively. 72% (89 children and 9 adults) of case group had high BLL (>10 μg/dL) while there were no cases of high BLL in controlled group. Lead concentration in soil vegetables and meat (fish and shellfish) exceeded the recommended standard	IQ scores of the case group (vicinity < 30 km from mining) were significantly lower than controlled group (82.7 ± 8.27 vs 96.14 ± 7.8)
	Youravong et al. (2006) [26]	Cross-sectional	Preschool to school child (6–10 years)	292	Blood lead	Children with BLL ≥ 10 μg/dL was 21%.	Lead exposure was associated with carries in deciduous teeth but not in permanent teeth (cariogenicity) in dose-response relationship.
	Youravong et al. (2013) [28]	Cross-sectional	Preschool to school child (6–10 years) (same population as [26])	120	Salivary and blood lead	The salivary lead level low correlated with blood lead level (R^2^ = 0.18, p = 0.05)	There was no association between salivary lead and dental caries.
**Mercury**	Umbangtalad et al. (2007) [46]	Case-controlled	School child (elementary student)	59 Part of larger study included adult	Hair and urinary mercury Environmental monitoring of mercury on water, sediment,	The urinary mercury was 15.82 and 9.95 µg/g creatinine in ‘involved’ and ‘not involved in mining activities’, respectively. Average Hg hair level in all schoolchildren (0.93 µg/g) with no significant difference between each group	The hazard quotient (HQ) based on the inorganic mercury exposure was < 1 (no risk).

*Age group reference: newborn: birth–1 month, infant: 1–23 months, preschool child: 2–5 years, school child: 6–12 years, adolescent: 13–18 years, child: birth–18 years. **Abbreviations**: BLL: blood lead level, cBLL: DMA: dimethylarsinic acid, EBLL: elevated blood lead level, Hb: haemoglobin, iAs: inorganic arsenic, MG: macroglobulin, MMA: monomethylarsonic acid.

**Table 4:** Epidemiological studies investigating air pollution exposure in Thai children.

Air pollution	Authors (published year)	Type of epidemiological study	Population (age)*	Sample size	Exposure assessment	Level measured	Comments

**Exposure studies**							

**Aeroallergens**	Lee et al. (2006) [61]	Cross-sectional	Dust from young child’s home	50	Endotoxin level and dust mite in dust from mattress of young child’s home	Endotoxin level in rural Thailand was higher than urban Singapore, however, dust mite allergen was higher in urban Singapore Endotoxin level was also prominent in agricultural area independent of farming environment	Cleaning practice and mattress type influenced the endotoxin level Air-conditioning also influenced the dust mite allergen level (positive correlation)
	Pumhirun et al. (1997) [60]	Cross-sectional	School child and adolescent (10–18 years)	Total 100 Part of larger study that included adult	Skin prick test (30 aeroallergens)	Allergic rhinitis patients were mostly sensitised to house dust mite (D. *farina*, D. *pteronyssinus*) and American cockroach.	85% of patients sensitive to house dust mite were positive to both D. pteronyssinus and D. farina (substantial cross-reactivity)
**Benzene**	Ruchirawat et al. (2005) [47]	Case-controlled	School child (10–12 years, male)	69	Benzene ambient air monitoring (personal) Blood benzene, and urinary t,t-MA levels	The benzene levels on the main roads ranged from 16.35 to 49.25 ppb. Benzene exposure were 4.71 ± 0.25 and 2.10 ± 0.16 ppb in exposure and control group, respectively.	School children in Bangkok were exposed in total Benzene more than control group (living outside the city)
**ETS**	Anuntaseree et al. (2008) [57]	Cross-sectional	Parent of infants	3,256	Questionnaires	Prevalence of at least one smoker in household was 47.2%, paternal smoking in the present of their infant was 35.1%, maternal smoking 0.3%	The significant association with paternal smoking was age (25–34 years), education less than secondary school, and Muslim father. This study was the part of the “Prospective Cohort Study of Thai Children”; PCTC
	Thongthai et al. (2008) [58]	Cross-sectional	Adolescent (15–19 years)	2,596 Part of larger study that included adult (≥15 years, total n = 28,248)	Questionnaires	Tobacco smoke affected 60% of population	Smokers were more likely to be male and older, but those exposed to secondhand smoke tend to be female and younger.
**PAHs**	Ruchirawat et al. (2005) [47]	Case-controlled	School child (10–12 years, male)	69	PAH ambient air monitoring (personal) Urinary 1-OHP	Total PAHs on the main roads ranged from 7.10 to 83.04 ng/m^3^. Total PAHs were 6.70 ± 0.47 and 1.25 ± 0.24 ng/m^3^ in exposure and control group, respectively	School children in Bangkok were exposed in total PAH more than control group (living outside the city)

**Exposure and health outcome assessment studies**							

**Benzene**	Buthbumrung et al. (2008) [51]	Case-controlled	School child (9–13 years, male)	171 (exposure 109, controlled 62)	Personal air and area sampling of benzene Blood benzene, urinary benzene and metabolite (MA) levels 8-OHdG in leukocyte and urine (oxidative DNA damage markers) DNA isolation and identification of metabolic genotype	Mean benzene level in ambient air at roadsides adjacent to the Bangkok schools were 17.75 ± 2.23 ppb and in rural school were 4.49 ± 0.59 ppb Level of 9-OHdG in exposure vs control group were 0.25 ± 0.02/105 dG vs 0.08 ± 0.06/105 dG	Children living in high traffic density areas were exposed to higher level of benzene than those living in rural area Benzene contribute to oxidative DNA damage.
	Navasumrit et al. (2005) [48]	Case-controlled	School child (12–14 years)	71 (exposure 41, controlled 30)		Bangkok school children (5.50 ppb) were exposed to significantly higher levels of benzene than provincial school children (2.54 ppb; p < 0.01). Increased DNA damage and a decreased DNA repair capacity in benzene exposed group were observed, compared to unexposed children.
	Ruchirawat et al. (2007) [49]	Case-controlled	School child (9–13 years, male)	184 (case 115, controlled 69)	Ambient benzene monitoring DNA strand breaks and DNA capacity Genetic polymorphisms of SGTs and CTP450	Benzene levels on children studying in Bangkok were significantly higher than (3.5 times) students studying in rural school (control group)	DNA strand breaks were significantly higher, but DNA repair capacity was significantly lower in Bangkok children. Genetic polymorphisms of GSTs and CYP450 enzymes were detected, involved in metabolism of benzene and PAHs, but no significant effects on the biomarkers of PAH exposure.
	Ruchirawat et al. (2010) [52]	Case-controlled	School child (9–13 years, male)	276 (city 165, rural 111) Part from larger study that includes adult	Ambient benzene and 1,3-butadiene, particle associated PAHs DNA-adduct of peripheral mononuclear white blood cells 8-OHdG in leukocytes DNA strand break and DNA repair capacity	School children in city and rural areas were exposed to 19.38 and 8.4 μg/m^3^ benzene respectively	Low level of benzene exposure, alone or concurrently with other carcinogens, resulted in early biological effect in the study.
**DEPs: urban air pollution**	Sriyaraj et al. (2008) [67]	Cross-sectional	School child (6–12 years)	511	Standard ISAAC questionnaires	Prevalence of cigarette smoking for mother was 4.5%, father was 49.3% and mother during the 1st year of child’s life was 5.3%	Potential environmental factors that were significantly positively correlated with the allergic disorders were diesel engine vehicles, antibiotic and paracetamol use, nuts consumed, contact with dogs and cats in the first year of life, contact with farm animal by mother while pregnant, and maternal cigarette smoking Prevalence of Rhinitis (24.3% vs. 15.8%), hay fever (23.2% vs. 13.9%), and atopic dermatitis (12.5% vs. 7.2%) were more common in urban than in suburban areas. The prevalence of asthma was not different between both areas (5.5%).
**ETS**	Sritippayawan et al. (2006) [59]	Case-controlled	Preschool child (0–5 years)	71	Urinary cotinine/creatinine, Questionnaires	28% of patients had history of in-house smoking. The median urinary cotinine level was 0.5 μg/mg Cr.	ETS exposure increased the risk of desaturation (SpO2 < 9%) in RSV-LRI but was not associated with RSV-LSI itself
**PAHs**	Pongpiachan et al. (2015) [56]	Cross-sectional	Preschool child (risk of PM2.5 intake in preschool children of age 0–5 years old)	Not applicable (risk assessment from environmental monitoring results)	PM2.5-bound PAHs	The average values of Σ3,4-ring PAHs and B[a]P equivalent concentrations in world urban cities were significantly much higher than those in samples collected from northern provinces during both sampling periods.	The cancer risk related to exposure through inhalation appears to be minor, while direct ingestion could potentially be a significant pathway for children due to their hand-to-mouth activities.
	Ruchirawat et al. (2006) [65]	Case-controlled	School child (10–12 years, male)	69 (case 44, controlled 25)	Ambient PAHs monitoring PAH-DNA adduct levels in lymphocytes	Ambient levels of PAHs are relatively high in Bangkok.	PAH-DNA adduct levels in lymphocytes were 5-fold higher in Bangkok
	Ruchirawat et al. (2007) [49]	Case-controlled	School child (9–13 years, male)	184 (case 115, controlled 69)	Ambient PAHs monitoring	PAHs of children studying in Bangkok were significantly higher than students studying in rural school (control group)	DNA strand breaks were significantly higher, but DNA repair capacity was significantly lower in Bangkok children Genetic polymorphisms of GSTs and CYP450 enzymes were detected, involved in metabolism of benzene and PAHs, but no significant effects on the biomarkers of PAH exposure.
	Ruchirawat et al (2010) [52]	Cross-sectional	School child (9–13 years, male)	276 (city 165, rural 111) Part from larger study that include adult	Ambient benzene and 1,3-butadiene, particle associated PAHs DNA-adduct of peripheral mononuclear white blood cells 8-OHdG in leukocytes DNA strand break and DNA repair capacity	School children in city and rural, respectively were exposed to: 1,3-butadiene–2.42 and 0.65 μg/m^3^ (city and rural) Total PAHs–4.13 and 1.18 (city and rural) B[a]P equivalents–1.50 and 0.43 ng/m^3^	Low level of benzene exposure, alone or concurrently with other carcinogens, resulted in early biological effect in the study populations.
	Tuntawiroon et al. (2007) [50]	Case-controlled	School child (8–13 years)	184 (case 115, controlled 69)	Urinary 1-HOP Ambient air monitoring DNA adduct level in peripheral lymphocytes	Concentration of urinary 1-HOP was significantly higher in Bangkok schoolchildren.	Bulky carcinogen-DNA adduct levels in peripheral lymphocytes were also significantly higher Significantly higher level of DNA strand breaks were significantly higher DNA repair capacity significantly lower in children in Bangkok.
**PM**	Aekplakorn et al. (2003) [62]	Cohort	School child (6–14 years, asthma and non-asthma)	175	PM10 ambient air monitoring Pulmonary function test Respiratory symptom questionnaires	A 10 µg/m^3^ increment was associated with changes in the highest FVC (–6.3 ml, 95% CI: –9.8, –2.8), FEV1 (–6.0 ml, 95% CI: –9.2, 2.7), PEFR (–18.9 ml.sec^–1^, 95% CI: –28.5, –9.3) and forced expiratory flow 25 to 75% of the FVC (FEF25–75%) (–3.7 ml.sec^–1^, 95% CI: –10.9, 3.5) in asthmatic children.	Declines in pulmonary function among asthmatic children were *associated with* increased in PM10
	Langkulsen et al. (2006) [64]	Cross-sectional	School child (10–15 years)	878 (completed PFT 722)	PM10 monitoring Questionnaire PFT	Prevalence of respiratory symptoms increased significantly in high polluted area [OR 2.44 (95% CI, 1.21–4.93) and 2.60 (1.38–4.91), in road side and general area, respectively].	Increased chronic respiratory symptom and impaired lung function in high-pollution area
	Preutthipan et al. (2004) [63]	Cohort	School child	133 (asthma 93, non-asthma 40)	PM10 air monitoring	PM10 levels exceeded 120 mg/m^3^ (Thai national standard at that time) for 14 days of 31 days records 4-hr average PM10 levels ranged between 46–201 mg/m^3^. PM10 levels exceeded 120 mg/m^3^ for 14 days. When PM10 levels were >120 mg/m^3^, the daily reported nasal irritation of asthmatic children was significantly higher than when PM10 levels were ≤ 120 mg/m^3^	PEFR did not change with different ambient PM10 levels in both groups. Elevated levels of PM10 concentrations in Bangkok affected respiratory symptoms of schoolchildren with and without asthma.
**SO2**	Aekplakorn et al. (2003) [62]	Cohort	School child (6–14 years, asthma and non-asthma)	175	SO2 air monitoring Pulmonary function test Respiratory symptom questionnaires	The ambient SO2 levels were relatively low except for a few days. The daily 24-hour mean SO2 concentrations were lower than the Thai ambient standards (300 µg/m^3^).	*No association* of pulmonary function decline with increased in SO2
**VOCs**	Aungudornpukdee et al. (2009) [54]	Cross-sectional	School child (6–13 years)	2,956	GIS Neurobehavioral test	The distance to industrial park from residential areas was not statistically different between children who had and had not motor coordination deficit.	The associated factors of visual-motor coordination deficit were gender, monthly parental income, children’s age, residential period, and household ETS
	Singkaew et al. (2013) [66]	Case-controlled	School child (4–11 years)	6 Part of larger study that included adult	Ambient air monitoring of VOCs	The average VOCs during 2006–2010 that exceeded Thai annual standards were 1,2-Dichloroethane, 1,3-Butadiene, and Benzene.	The lifetime cancer and non-cancer risk in all high-risk group including children were in acceptable range based on the US EPA health risk assessment.

*Age group reference: newborn: birth–1 month, infant: 1–23 months, preschool child: 2–5 years, school child: 6–12 years, adolescent: 13–18 years, child: birth–18 years. **Abbreviations**: Cr: creatinine, CYP450: cytochrome P450, DEP: diesel exhaust particle, ETS: environmental tobacco smoke, FEV1: forced expiratory volume at 1 second, FVC: forced vital capacity, GIS: Geographical Information System, GSTs: glutathione-S-transferases, MA: muconic acid, OR: odds ratios, PAHs: Polyaromatic hydrocarbons, PEFR: peak expiratory flow rate, PFT: pulmonary function test, PM: Particulate matter, SO2: Sulphur dioxide, t,t-MA: t,t-muconic acid, 1-HOP: 1-hydroxypyrene, VOCs: volatile organic compounds, 8-OHdG: 8-oxo-7, 8-dihydro-2’-deoxyguanosine, 95% CI: 95% confidence interval.

### Pesticides

There were the 12 full-text articles available that investigated pesticide exposure in Thai children (Table 2), three of which measured both exposure and subsequent health effects in the same cohorts. A number of pesticide metabolites were detected in the urine/serum of Thai children, including those of organochlorine [8,9], organophosphate [10,11,12,13,14,15], parathion [16], pyrethroid [11,15,17], glyphosate [18], and paraquat [18]. These include pesticides that were officially banned prior to the study periods [8,9,16].

Factors associated with pesticide exposure included living close to agricultural farmland, frequency of agricultural field visit agricultural activities, insecticide use in the home, household environment, and child-specific factors (such as frequency of pesticides used on farm, being a member of a rice farming family, proximity to rice farm, being with parent on rice farm, playing on rice farm, parentally observed dirt on body) [19]. In addition, Liu et al. demonstrated the indirect transfer of pesticide from the farmer to child through contamination of the home environment [14]. The health outcomes associated with organochlorine (dichlorodiphenyltrichloroethane; DDT) or organophosphate (diethyl phosphate; DEP and diakylphosphates; DAP) were altered thyroid function/hormone levels [8] or neurotoxicity [12], respectively. The third study that investigated health outcomes showed no association between organophosphate or pyrethroid exposure and neurobehavior in school children [11].

### Heavy Metal

There were 21 full-text articles available that investigated heavy metal exposure in Thai children (Table 3), nine of which measured both exposure and subsequent health effects in the same cohorts (lead, three articles; arsenic, four articles; cadmium, one article; and mercury, one article).

The current sources of lead exposure in Thai children (following the ban on leaded fuel) appear to be para-occupational and related to household location and activities [21,22,23,24,25,26,27,28,29,30]. The high risk areas of lead exposure were boatyard and shipyard factories or locations of former mining and smelting. Maharachpong et al. demonstrated lead contamination in coastal communities connected with boat repair and boat building activities [31]. Increased levels of lead in household dust were observed in households with a member that worked on boat repair (either in boatyards or at home). Similarly, Pusapukdepob et al. demonstrated lead contamination from mining, where the waste from lead mining that was discharged directly into a stream near Klity village in 1998 was still present in the sediment over a decade later [29]. The risk factors for elevated blood lead levels were anemia, large families, male gender, age under two years, access to lead-acid batteries, and residence in a house with peeling paint [21,22,30,32,33]. The health outcomes associated with chronic lead exposure in Thai children were cognitive deterioration and dental caries [26,29].

The studies on arsenic exposure in Thai children were all focused in the Ron Pibul district, Nakorn Srithammarat province, Southern region of Thailand. Here, former tin mining and smelting activities had led to the contamination of drinking water, soil and some of the food chain with arsenic [34,35,36]. Furthermore, the level of arsenic exposure was exacerbated by the children’s risk behavior, such as playing with soil and no hand-washing before eating [34]. The health outcome associated with arsenic exposure in utero and through childhood was increased cancer risk, where hypomethylation of inflammatory genes (*COX2, EGR1*, and *SOC3*) positively correlated with levels of 8-nitroguanine, increased DNA damage and reduce DNA repair capacity [35,36].

Likewise, cadmium exposure in the Northern region appeared to be mainly a result of tin mining activity leading to contamination of land, water, and eventually the staple food (rice grown in contaminated soil) [37]. However, the level of cadmium exposure in Thai children appeared to be decreasing due to cessation of mining activity and subsequent declining environmental contamination [38]. It is therefore hoped that the renal effects associated with cadmium exposure the in local Thai children will also continue to decrease [37,39]. Indeed, Swaddiwudhipong et al. have reported no association between cadmium exposure and blood pressure in children [37].

Finally, children attending an elementary school near a small-scale gold mine in Phichit demonstrated elevated urinary inorganic mercury levels. However, the hazard quotient (HQ) based on the inorganic mercury exposure was <1 in these school children [40], suggesting they are not at risk of nephrotoxicity from inorganic mercury at this level of exposure [41].

### Air Pollution

The majority of the 17 full-text articles available investigated exposure to outdoor air pollution, and secondhand or environmental tobacco smoke (ETS) (Table 4). The source of outdoor air pollution depended on the geographical area, and included traffic-related pollution in inner cities, petrochemical factories in coastal provinces, coal power plants and agricultural burning activities in the northern region of Thailand.

Traffic-related and automotive emissions, including diesel exhaust particles, have been reported in urban areas and linked to both cancer and non-cancer risks [47,48,49,50,51,52]. Studies of petrochemical industrial estates have focused on volatile organic compounds (VOCs), particularly benzene and polyaromatic hydrocarbons (PAHs), demonstrating associations with both occupational and environmental health in central Thailand [48,52,53,54,55]. For example, exposure to 1,3-butadiene and PAHs in children living close to petrochemical industries was associated with increased cancer risk [52].

Outdoor biomass burning activities (including forest fires, agricultural, and refuse burning) appear to be a major sources of air pollution in Northern Thailand, potentially creating PM2.5-bound PAHs [56]. However, there was limited evidence of related health outcomes (either respiratory or non-respiratory diseases such as cancer risk) [56].

There have been two large studies assessing the exposure of Thai children to secondhand smoke inhalation: 1) the Prospective Cohort Study of Thai Children (PCTC) covering Bangkok and across Thailand (3,256 participants, all were parents of infants aged less than 1 year) [57] and 2) the fifth-round census of the Kanchanaburi Demographic Surveillance System (KDSS) covering the Kanchanaburi province (28,248 participants, 2,596 children of 15–19 years) [58]. Anuntaseree et al. reported that the prevalence of at least one smoker in a household was 47.2%, paternal smoking in the presence of an infant was 35.1%, and maternal smoking was 0.3% [57]. Paternal smoking was significantly associated with an age of 25–34 years, education lower than secondary school, and a Muslim father [57]. Thongthai et al. reported the prevalence of secondhand smoke exposure in 60% of the Kanchanaburi population (24.6% and 1% prevalence in male and female teenagers, respectively) [58]. The smokers in this report were more likely to be older and male, while those exposed to secondhand smoke tended to be younger and female. Despite these surveys, there was only one study that investigated both ETS exposure and health outcomes in Thai children, which demonstrated an association between ETS exposure and an increase of the severity of respiratory syncytial virus (RSV) infection in young children [59].

There was one study that examined sensitization in Thai children suffering from allergic rhinitis, reporting that the predominant aeroallergen to which patients were sensitized in Bangkok was house dust mite [60]. A subsequent exposure study collected dust from the mattresses of young children in Thailand and Singapore [61]. Dust mite allergen in rural Thailand was less than in urban Singapore and positively correlated with air-conditioning use, while the endotoxin level in rural Thailand was higher (particularly in agricultural areas) compared to Singapore. The cleaning practices and type of mattress were associated with endotoxin levels.

### Specific Geographical Area of Health Effect Related to Environmental Exposure in Thailand

Focusing on the evidence of health effects related to environmental exposure in particular geographical areas in Thailand (Table 5), six major health effects were identified: neurotoxicity (IQ and neurodevelopment), respiratory impairment, renal effect, endocrine disorder, allergic diseases, and cancer risk (probably via DNA damage and impaired DNA repairing). The IQ deterioration from chronic lead exposure was reported in Klity village, Kanchanaburi (central region) [29]. The neurodevelopmental effect was associated with *in utero* exposure to organophosphate in Amnatchareon, Nakhon Sawan and Kanchaburi [12]. In contrast, in the greater Bangkok area, which was rice farming area, organophosphates, chlorpyrifos, and pyrethroid were detected but there was no statistically significant association with neurobehavioral problems in school age children [11]. Respiratory effects, such as declined pulmonary function or chronic respiratory symptoms, were associated with air pollution (PM) in the Maemoh district, Lampang, and Bangkok [62,64]. Early renal effects from chronic cadmium exposure, reduced cord serum T4 from organochlorine exposure, and allergy risk of allergic rhinitis and atopic dermatitis from air pollution (DEPs) were found in Tak and Chiang Mai, respectively [37,39,67]. Increased cancer risk and DNA damage, and decreased DNA repair capacity, were detected in Bangkok and the Ron Phibul district, Nakorn Sri Thammarat. However, the culprit hazards were different: air pollution (benzene, traffic-related PAH) in Bangkok, and arsenic in the Ron Phibul district [34,35,47,48,49,50,51,52,59,60,63,65,68].

**Table 5:** Health effects related to environmental exposure of particular geographical areas in Thailand.

Region	District, province	Hazards	Health effects association/health risk		References

			Yes	No	

**Northern**	Umpang, Tak	Lead	NA	NA	[22,43,44]
	Mae Sot, Tak	Cadmium	Early renal effect	NA	[37,39]
	Phonom Pha, Phichit	Mercury (inorganic)	NA	Hazard quotient	[46]
	Mae Moh, Lampang	Air pollution (SO2, PM)	Decline of pulmonary function (PM)	Decline of pulmonary function (SO2)	[62]
	Chiang Mai	Air pollution (DEPS)	Allergic diseases (rhinitis, atopic dermatitis)	NA	[67]
	Mae rim, Chiang Mai	Organochlorine	Negative association of cord serum total T4 levels with DDT metabolites	NA	[8]
**Northeastern**	Amnatchareon	Organophosphate	Cognitive and motor development in infant	NA	[12]
**Central**	Bangkok	Lead (some areas)	NA	NA	[30,32]
		Air pollution (PM, methane, traffic related PAHs, benzene aeroallergen, ETS)	Respiratory symptom Impaired pulmonary function Increased severity of RSV infection (ETS) Increased PAH-DNA adduct levels Increased DNA damage and decreased DNA repair capacity Increased bulky carcinogen-DNA adduct levels Cancer risk Allergic rhinitis (aeroallergen sensitization)	Upper respiratory infection	[47,48,49,50,51,52,59,60,63,64,65,68]
	Greater Bangkok (rice farming)	Organophosphates, chlorpyrifos, and pyrethroid	NA	Neurobehavioral in school age children	[11]
	Klity village, Kanchanaburi	Lead	Dental caries, IQ deterioration	NA	[29]
	Kanchanaburi	Organophosphate	Cognitive and motor development in infant	NA	[12]
	Map Ta Phut, Rayong	VOCs (petrochemical industrial area)	NA	Visual-motor coordination deficits Health risk (cancer and non-cancer)	[54,66]
	Nakhon Sawan	Organophosphate	Cognitive and motor development in infant	NA	[12]
**Southern**	Singhanakorn district, Songkhla	Lead	Dental caries	Dental morphologic change	[25,26]
	Ron Phibul district, Nakhon Sri Thammarat	Arsenic	Increased DNA damage and decreased DNA capacity Cancer risk Hypomethylation of inflammatory genes (*COX2, EGR1, and SOC3*) positively correlated with levels of 8-nitroguanine.	NA	[34,35,36]

**Abbreviations:** DDT: Dichlorodiphenyltrichloroethane, DEPs: diesel exhaust particles, ETS: environmental tobacco smoke, IQ: intelligent quotient, NA: not applicable, PAH: polyaromatic hydrocarbons, PM: particulate matter, SO2: Sulphur dioxide, VOCs: volatile organic compounds.

## Discussion

### Children’s Environmental Health in Thailand: Past Issues

Before the 21st century, Thailand was classified as a lower-middle income country. Industrialization had begun, but with less awareness of the environmental impact. This led to the major pollution of air, water and soil, which in turn had a negative effect on the population’s health. A key legislative change that positively impacted environmental health in Thailand (including children’s health) was the lead-free gasoline policy in 1993. This policy gradually reduced lead in fuel from 1984 onwards. Unleaded petrol was introduced in 1991, and fuel containing lead was completely phased out in 1993. The resulting impact was demonstrated by the improvement of average air quality in Bangkok in 1998. Studies showed that over a one-month period the lead level in ambient air was below the standard limit of 1.5 μg/m^3^. In addition, the blood levels of lead in children living in urban areas also showed a decline. By 2002, blood lead levels in Bangkok children had decreased compared to those levels in neonates and school children measured in 1989 [32,69].

### Children’s Environmental Health in Thailand: Current Issues

For two decades now, sanitization has improved and the mortality rate from infectious diseases in Thailand has decreased. However, there is still an imbalance between industrialization and environmental protection. Rapid globalization and urbanization have led to the emergence of modern hazards, including pesticides [8,10,11,12,13,14,15,16,17,18,19,20,35,70,71,72]; heavy metals such as lead [21,22,23,24,25,26,27,28,30,31,42,43,44,69], arsenic [34,36], and cadmium [37,39]; and air pollution such as traffic-related emissions in urban areas [47,48,49,50,51,52,63,64,65,67], VOC emissions from petrochemical industries [48,54,66,73], environmental tobacco smoke [57,58,59], particulate matter from fires/burning [56,67], and toxic gases from power plants [62].

### Pesticides

Traditional pest management (with chemical pesticides) in Thailand seems to be a more common practice than integrated pest management [13]. Pesticides, including insecticides, herbicides, fungicides, rodenticides, fumigants, and repellants, are widely used for both agricultural and domestic management. Each product can be subdivided according to chemical structure, which reflects mechanisms of action and toxicity. Organophosphate, carbamate, and pyrethroid insecticide use is currently allowed in Thailand. In contrast, the use of the organochlorine dichlorodiphenyltrichloroethane (DDT) was banned in 1989. Between 1949 to 1989, DDT was used as a pest control in farming and to control the spread of malaria by limiting the number of mosquitoes. However, exposure to DDT was detected via biomonitoring in agricultural areas a number of years later [8,9]. This result may be a result of the bioaccumulative and persistent nature of DDT, or it may reflect illegal use. DDT acts as an endocrine disruptor and showed a negative association with thyroid hormones in the cord blood of newborn Thai children [8]. Chronic exposure in children can occur via air, soil, dust, food, and through direct exposure to residential/garden agricultural products or product residues. Of highest risk are children and pregnant women who live in agricultural areas or live with agriculturists [74]. Contact with potentially contaminated dirt/soil, hand-to-mouth activity, and walking/playing on the farm all represent possible means of exposure in children. These children also encounter more accidental exposures as a result of products being taken home and spilled [10,12,13,14,19,20]. However, only two studies have evaluated subsequent health effects in Thai children, both of which focused on neurodevelopment. The first, a case-controlled study in school children, showed no association between organophosphate (dialkylphosphates; DAPs) or pyrethroid exposure and neurobehavior [11]. In contrast, the second, which looked at a birth cohort in an unspecified agricultural area (50% of participants were farm workers), demonstrated a negative association between organophosphate exposure (diethyl phosphate) and cognitive and motor development of infants at five months of age [12]. This discordance may be due to the difference in age group, neurobehavioral assessment, and the timing/level of pesticide exposure. Furthermore, there is increasing evidence from other studies outside Thailand supporting the neurotoxicity of low-dose chronic organophosphate exposure [75,76].

### Heavy metals

Although the lead-fuel ban has had a major impact on lead exposure in Thai children, other sources of environmental lead contamination remain. These include former mining areas, shipyards, lead-based paints, and the use of car batteries in the household. Indeed, the extensive use of pumbopumpic oxide (Pb_3_O_4_) in boat repairing activities in the southern region of Thailand has caused lead contamination of the surrounding air, soil and dust [31]. As a result, lead exposure in children living in such contaminated environments is unavoidable.

Chronic arsenic exposure in Thai children has been detected in the vicinity of the former tin mining and smelting activities in the Ron Pibul district of the Nakorn Srithammarat province in the Southern region of Thailand. There were more than 1,000 reported cases of arsenicosis in 1987 and mining was eventually banned in 1994, resulting in arsenopyrite and pyrite-rich waste piles [34,35]. Arsenic exposure from this waste remains, as demonstrated by the detection of biomarkers of exposure and disease reported in multiple studies [34,35,36]. The major health risks associated with arsenic exposure are carcinogenic [77]. Indeed, early life arsenic exposure has been associated with DNA damage (8-nitroguanine or 8-OHdG formation) and activation of inflammatory genes (*COX2, EGR1* and *SOC3*) through a proposed epigenetic mechanism involving hypomethylation and increased mRNA expression [35,36,45].

Chronic cadmium exposure in Thai children has not been widely studied and the current source of exposure appears to be limited to the former zinc mining areas of the Mae Sot district, Tak province, northern Thailand. This zinc mine was established in 1977 and environmental cadmium contamination was first demonstrated in 1998 [78]. Most of the adverse health effects were identified in adults. However, there were two studies reporting cadmium exposure in children and potential adverse effects on renal impairment [37,39]. Although cadmium environmental contamination appeared to decline after cessation of the zinc mining operation, other sources of environmental cadmium exposure, such as second- or third-hand smoking exist and should be considered.

### Air pollution

Like other developing countries, air pollution in Thailand is prominent in inner cities and industrialized provinces, with the specific types of air pollution depending upon the geographical area [2]. The main sources of outdoor air pollution in Thailand are: traffic in cities and industrial provinces (VOCs, benzene, PAHs, and particulate matter); petrochemical industries in coastal areas (VOCs, benzene) areas; and coal power plant and open biomass burning in the northern region of Thailand (particulate matter and PAH). In contrast, ETS, which can be both an indoor and outdoor air pollutant, is more generally prevalent across Thailand. Approximately 50% of the Thai population has contact with secondhand smoke, which is similar to the global secondhand tobacco smoke prevalence [79]. Likewise, house-dust and/or cockroach allergens represent another more widespread indoor air exposure across Thailand [80]. Sensitization to these allergens is very common in Asian countries, including Thailand [60,61]. Some sources of indoor air pollution are also linked with outdoor air pollution and children tend to spend more time in indoors than outdoors. For example, children can be exposed via direct transfer of chemicals from parents working in agriculture [14]; dust in households living close to boat repair yards contain higher levels of lead [31]; and endotoxin levels are higher in households located in agricultural areas [61]. The latter observation may be linked to the hygiene hypothesis and might explain the lower prevalence of allergic disease in Thailand compare to other Western countries [81]. The health outcomes associated with air pollution in Thai children were well-established effects on the respiratory system, and some genotoxic and potentially carcinogenic outcomes [47,49,51,63,64,65,67].

It is also important to note that those studies that demonstrated no association between environmental exposures and health outcomes in Thai children are also very useful from both a scientific and policy perspective. They highlight those outcomes that are less likely to be linked with environmental exposures, identify more resistant life stages, may help set thresholds of exposure**/**toxicity and focus the limited resources on the most important issues.

### Children’s Environmental Health in Thailand: Proposed Future Direction of Research

To further improve children’s environmental health in Thailand, we suggest that: 1) future epidemiological and molecular studies combine exposure and health outcome assessment in a prospective design and incorporate state-of-the-art technologies where appropriate, 2) regular monitoring of the levels of current hazards such as pesticides, heavy metals and air pollutants should be considered, and 3) vulnerable populations such as pregnant women and children be given greater attention. Such studies would help to identify the key pollutants associated with children’s health in Thailand, and develop policy changes that will achieve the greatest impact.

## Conclusions

Environmental health in Thailand is improving in terms of awareness, expanding knowledge, and increasing environmental conservation. However, pesticide, heavy metal, and air pollution exposures are still major issues that can affect the health of the next generation. Further advancement of children’s environmental health in Thailand now requires a greater understanding of the links between exposures and outcomes, education of the public and policymakers, development of policies in environmental health, improved regulations, and enhanced coordination between organizations.
